# Correction: Largest global shark biomass found in the northern Galápagos Islands of Darwin and Wolf

**DOI:** 10.7717/peerj.1911/correction-1

**Published:** 2018-02-01

**Authors:** Pelayo Salinas-de-León, David Acuña-Marrero, Etienne Rastoin, Alan M. Friedlander, Mary K. Donovan, Enric Sala

**Affiliations:** 1 Department of Marine Sciences, Charles Darwin Research Station, Puerto Ayora, Galapagos Islands, Ecuador; 2 Pristine Seas, National Geographic Society, Washington, D.C., USA; 3 Fisheries Ecology Research Lab, University of Hawai’i at Manoa, Honolulu, HI, USA

Corrigendum:

After publication the authors noticed that the corresponding author's surname was listed incorrectly. The author list and author contributions have been amended so that Pelayo Salinas de León is now listed as Pelayo Salinas-de-León. 

