# Supplementary material for: Largest global shark biomass found in the northern Galápagos Islands of Darwin and Wolf
Source: PeerJ. 2016 May 10;4:e1911. doi: 10.7717/peerj.1911 (PMC4867720; doi:10.7717/peerj.1911)
Supplement: Supplemental Information 3 [file peerj-04-1911-s003.docx]

**Supporting information**

**Contents:**

- Figure_S1. Sharks size frequency distribution between DOVs and UVC
- Table_S1. Fish species most responsible for dissimilarity between wave exposures.

**Figure_S1.** **Sharks size (mm) distribution recorded by DOVs and UVC.** Data was obtained from 69 transects at 7 study sites across Darwin and Wolf Islands.


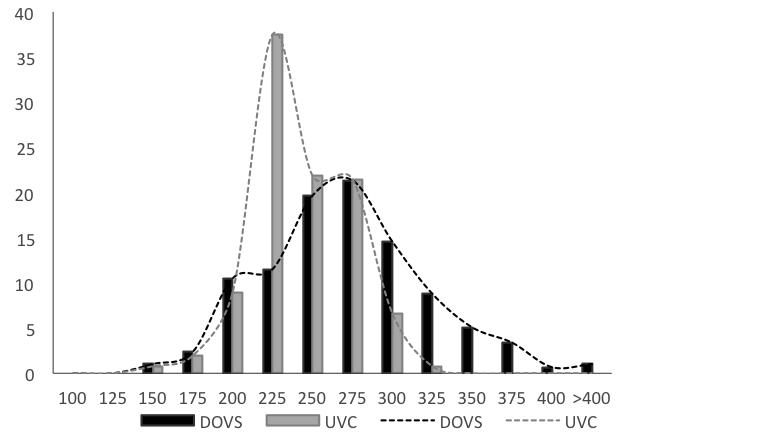


**Table_S1**. Fish species most responsible for the dissimilarity between wave exposures (SE vs. NW) based on Similarity of Percentages (SIMPER) analysis. Values are biomass (t ha^-1^). Diss. – average dissimilarity. SD – Standard deviation. Contrib% – percent contribution to dissimilarity. Cum. % – Cumulative dissimilarity.

| Species | SE | NW | Diss. | SD | Contrib% | Cum.% |
| --- | --- | --- | --- | --- | --- | --- |
| *Sphyrna lewini* | 18.44 | 0 | 35.07 | 1.13 | 38.51 | 38.51 |
| *Paranthias colonus* | 2.49 | 0.67 | 18.72 | 0.96 | 20.55 | 59.06 |
| *Carcharhinus galapagensis* | 3.09 | 0 | 16 | 0.64 | 17.57 | 76.63 |
| *Caranx melampygus* | 1.06 | 0 | 2.52 | 0.28 | 2.77 | 79.4 |
| *Taeniura meyeni* | 0.01 | 0.14 | 2.24 | 0.22 | 2.46 | 81.86 |
| *Prionurus laticlavius* | 0.03 | 0.1 | 2.13 | 0.41 | 2.33 | 84.2 |
| *Kyphosus analogus* | 0 | 0.15 | 1.97 | 0.2 | 2.16 | 86.36 |
| *Lutjanus novemfasciatus* | 0.05 | 0.03 | 1.35 | 0.28 | 1.48 | 87.84 |
| *Scarus ghobban* | 0 | 0.05 | 1.2 | 0.22 | 1.32 | 89.16 |
| *Holacanthus passer* | 0.03 | 0.07 | 0.99 | 0.52 | 1.09 | 90.25 |
